# Complexity and Barriers to Vision Care: A Narrative Review Informed by a Mobile Eye Program

**DOI:** 10.3390/ijerph22121880

**Published:** 2025-12-18

**Authors:** Valeria Villabona-Martinez, Anne Schulman, Bharadwaj Chirravuri, Kerollos Kamel, Paula A. Sepulveda-Beltran, Zeila Hobson, Evan L. Waxman

**Affiliations:** 1 Department of Ophthalmology, University of Pittsburgh Medical Center, Pittsburgh, PA 15219, USA; villabonamartinev@upmc.edu (V.V.-M.); sepulvedabeltranp@upmc.edu (P.A.S.-B.); hobsonz@upmc.edu (Z.H.); 2 Department of Ophthalmology, University of Pittsburgh School of Medicine, Pittsburgh, PA 15260, USA; schulman.anne@medstudent.pitt.edu (A.S.); chirravuri.bharadwaj@medstudent.pitt.edu (B.C.); kamel.kerollos@medstudent.pitt.edu (K.K.)

**Keywords:** health disparities, implementation science, patient navigation, ophthalmology, barriers to care

## Abstract

**Highlights:**

**Public health relevance—How does this work relate to a public health issue?**

- Preventable vision loss disproportionately affects underserved communities due to both patient-level barriers (e.g., language, insurance, financial hardship) and system-level failures that delay or interrupt medically necessary, time-sensitive ophthalmic care.
- By examining real-world cases alongside the literature, this work highlights how health system rigidity, misclassification of ophthalmic conditions as “non-urgent,” and fragmented coordination contribute to inequitable access to essential eye care.

**Public health significance—Why is this work of significance to public health?**

- Vision impairment directly impacts functional independence, chronic disease outcomes, and quality of life; preventing avoidable delays in time-sensitive eye care is therefore a critical public health priority.
- This manuscript demonstrates that improving equity requires attention not only to individual barriers but also to the structural and organizational dynamics that shape care pathways, particularly when the system fails to recognize or prioritize medically necessary, time-sensitive ophthalmic treatments.

**Public health implications—What are the key implications or messages for practitioners, policy makers and/or researchers in public health?**

- Efforts to improve vision-care equity must combine patient-centered strategies (navigation, communication, support for insurance and transportation) with system-level improvements such as adaptive scheduling, cross-agency coordination, and prioritization frameworks that reflect both clinical urgency and the time-sensitive nature of many ophthalmic conditions.
- Integrating complexity-informed approaches can help health systems recognize early signs of system strain, respond flexibly to patient needs, and ensure that medically necessary, time-sensitive ophthalmic procedures are not delayed simply because they are not classified as emergencies, thereby preventing avoidable vision loss in vulnerable and non-vulnerable populations.

**Abstract:**

**Purpose**: To describe structural and systemic barriers to ophthalmic care experienced by underserved patients, particularly those facing language obstacles, immigration-related constraints, limited insurance coverage, financial hardship, and navigation challenges in an urban setting, and to examine these barriers through a complexity-informed lens. **Methods**: We conducted a narrative literature review focused on healthcare disparities, patient navigation, complexity in care delivery, and time-sensitive prioritization frameworks in ophthalmology. Findings were integrated with case vignettes drawn from Eyes on Wheels (EOW), a mobile eye care initiative that provides no-cost examinations at Federally Qualified Health Centers (FQHCs) and free clinics. Cases were identified through routine clinical documentation and used to illustrate how structural barriers described in the literature manifest in real-world care pathways. **Results**: Three recurring system-level issues were identified across EOW encounters: (A) misclassification of medically necessary, time-sensitive ophthalmic care as “non-urgent”; (B) patient disengagement driven by cumulative structural and logistical barriers; and (C) failures that arise when the healthcare system, functioning as a complex adaptive system (CAS), is unable to adapt to patients’ and systems’ changing circumstances. A review of the literature confirmed that these patterns reflect widely documented challenges faced by underserved urban populations. Three EOW case vignettes, selected from seven patients identified in 2024, are presented as illustrative examples of these systemic patterns. **Conclusions**: Addressing inequities in eye care requires an approach that recognizes how many parts of the healthcare system interact and affect a patient’s ability to receive timely treatment. Vision loss is often the preventable result of systems that are rigid, fragmented, or unable to adapt to a patient’s circumstances. Improving outcomes will require flexible care models, such as mobile clinics, paired with strong institutional support, patient-centered navigation, and consistent assessment of social needs and barriers to care. Sustained progress will depend on collaboration across organizations, adaptable leadership, and policies that respond to the real-world situations in which patients live.

## 1. Introduction

Eyes on Wheels (EOW) is a long-standing student-run mobile eye clinic program operated by the University of Pittsburgh and supported by the Eye and Ear Foundation of Pittsburgh. Since 2005, it has delivered comprehensive eye exams, without charge, to individuals facing barriers to care. The program conducts approximately three outreach missions each month at indigent care sites, primarily Federally Qualified Health Centers (FQHCs) and free clinics, serving 10 to 15 patients per event. When further treatment is required, patients are referred to the UPMC Vision Institute (VI).

Despite longstanding efforts, many EOW patients experience challenges obtaining follow-up care. These obstacles are consistent with well-documented barriers reported in the literature, including language discordance, limited insurance coverage, financial hardship, administrative complexity, and difficulties navigating fragmented systems [1,2,3,4,5]. EOW has implemented strategies such as insurance support, charity care pathways, navigation assistance, and close collaboration with FQHC partners; however, uniform success in obtaining timely follow-up care remains elusive. Program experience has shown that beyond traditional access barriers, more subtle structural and systemic issues often prevent care.

First among these is the misunderstanding of medically necessary, time-sensitive (MeNTs) ophthalmic care as “optional” or “elective”. Conditions such as glaucoma, diabetic retinopathy, macular degeneration, and cataracts are not immediately life-threatening, yet delays in evaluation or treatment can lead to irreversible vision loss and profound downstream effects on health and independence [6,7,8,9,10,11,12,13]. Existing insurance, financial assistance, and scheduling systems, not designed to recognize this time sensitivity, often contribute to harmful delays.

Second, communication breakdowns between patients and the many entities involved in their care, primary care providers, eye care providers, insurance agencies, governmental offices, and community organizations, can result in “patient disengagement” [14,15,16,17]. When compounded with day-to-day challenges such as completing paperwork, managing work schedules, or accessing medications, patients may stop participating despite initial willingness. This disengagement is frequently interpreted as a lack of motivation or responsibility, when it more accurately reflects cumulative system overload.

Finally, healthcare operates as a complex adaptive system (CAS). Even under ideal circumstances it is dynamic, nonlinear, and composed of many interacting agents: patients, providers, policies, technologies, and administrative structures [18,19,20,21]. For underserved populations, this complexity is intensified. Rigid processes, fragmented communication, and limited adaptability can cause small breakdowns to cascade, ultimately resulting in missed appointments, delayed diagnoses, or preventable loss of vision.

These observations from the EOW program form the basis of the present work. We conducted a narrative review to contextualize and expand upon three recurring themes identified through program experience: (A) misclassification of medically necessary, time-sensitive ophthalmic care as “non-urgent”; (B) patient disengagement driven by cumulative structural and logistical barriers; and (C) failures that arise when the healthcare system, functioning as a complex adaptive system (CAS), is unable to adapt to patients’ and systems’ changing circumstances. The following sections integrate evidence from the literature with illustrative case vignettes to demonstrate how these systemic patterns manifest in real-world care pathways.

## 2. Materials and Methods

### 2.1. Study Design and Ethics

This work began with the identification of patient cases within the Eyes on Wheels (EOW) mobile eye care program. Three cases seen during EOW encounters in 2024 revealed recurring barriers related to access, navigation, insurance classification, and continuity of care. These barriers informed the development of the subsequent narrative review, which synthesized literature relevant to the patterns observed in practice. Case information was drawn from routine clinical documentation and program navigation notes, and follow-up was monitored as part of standard care coordination. Because the cases were fully de-identified and derived retrospectively from routine operations and quality-improvement activities, they did not constitute human subjects research and therefore did not require IRB review, as confirmed through consultation with the institutional IRB office.

### 2.2. Literature Search and Thematic Synthesis

To contextualize the barriers first identified through case review, we conducted a targeted narrative search in PubMed, Web of Science, and Google Scholar. Six focused searches (Appendix A) covered literature from 2000–2025 on healthcare disparities, patient navigation, vision insurance, social determinants of health, complexity in healthcare, and definitions of urgency in ophthalmology.

More than 2000 titles were screened, followed by abstract and full-text review by VVM, AS, BC, KK, and PASB. Articles were included if they addressed structural or system-level barriers to eye care, ophthalmic disease diagnosis or management, or concepts relevant to complexity science, and if they aligned with at least one of the three thematic areas. Studies were excluded if they lacked relevance to ophthalmic access, focused solely on clinical or technical outcomes, or reflected health system contexts not comparable to the U.S. setting.

Through iterative qualitative synthesis of the literature and program experience, three themes consistently emerged: (A) misclassification of medically necessary, time-sensitive ophthalmic care as “non-urgent”; (B) patient disengagement driven by cumulative structural and logistical barriers; and (C) failures that arise when the healthcare system, functioning as a complex adaptive system (CAS), is unable to adapt to patients’ and systems’ changing circumstances.

Three cases from seven identified patients were purposively selected to illustrate these themes.

## 3. Results

### Themes, Cases, and Complexity-Informed Strategies

Analysis of the seven Eyes on Wheels (EOW) cases identified in 2024 revealed three recurring barriers that consistently interfered with patients’ ability to obtain timely ophthalmic care. These barriers were then explored in the narrative review, which revealed that while elements of these patterns are addressed across the published literature, they have not been synthesized and collectively illustrated. The three themes that emerged were:

(A) Misclassification of medically necessary, time-sensitive ophthalmic care as “non-urgent”
(B) Patient disengagement driven by cumulative structural and logistical barriers
(C) Failures that arise when the healthcare system, functioning as a complex adaptive system (CAS), is unable to adapt to patients’ and systems’ changing circumstances.

From the seven identified patients, three vignettes were purposively selected to illustrate each of these themes. These cases are not intended to represent all patient groups but to demonstrate how the systemic issues recognized in clinical practice are reflected in broader evidence on access to eye care.

*A*. ***Misclassification of medically necessary, time-sensitive ophthalmic care as “non-urgent”***

Triage, a concept originally applied on the battlefield and adapted to the emergency department, prioritizes care for patients with immediate threats to life or function. It is intended to sort and prioritize the injured or ill according to their need for emergency care [22]. A focus solely on emergencies is not always appropriate outside of the emergency department.

This became especially evident during the COVID-19 pandemic, when healthcare systems were forced to allocate limited resources while maintaining continuity of care. To address this challenge, Prachand et al. introduced the “Medically Necessary, Time-Sensitive” (MeNTS) scoring system in 2020 to guide surgical prioritization based on urgency, patient factors, and resource demand [23]. Teja et al. subsequently adapted MeNTS for ophthalmology [24], emphasizing that many ophthalmic procedures are neither emergent nor truly elective.

Recent evidence further highlights the potential value of structured approaches. A 2024 analysis of nearly 3000 general surgery cases found that MeNTS-based scheduling did not reproduce racial or ethnic disparities in time-to-surgery, suggesting that standardized tools may reduce bias associated with discretionary decision-making [25]. However, neither the original nor ophthalmology-adapted MeNTS frameworks incorporate specific social determinants of health (SDOH). For underserved patients, factors such as insurance instability, language discordance, transportation barriers, and limited navigation support can dramatically increase vulnerability to delayed care [2,3,4,5,26,27,28,29,30,31,32,33,34].

- Glaucoma progresses silently and irreversibly without consistent monitoring, medication use, or timely surgery [6,7,8].
- Proliferative diabetic retinopathy (PDR) requires timely injections, laser therapy, and coordinated systemic care to prevent rapid deterioration [9,10].
- Neovascular AMD can lead to permanent central vision loss, even with short lapses in injection intervals [11,12].
- Visually significant cataract is reversible but can cause profound functional disability, affecting mobility, work, and the ability to manage other health needs [13].

The requirement for treatment for these conditions --unlike ocular trauma or a retinal detachment—is not emergency care. Treatment is not however “optional” and timely treatment is critical [6,7,8,9,10,11,12,13,24].

Table 1 summarizes how major ophthalmic diseases differ in their time sensitivity, vulnerability to systemic barriers, and potential consequences of delayed care.

Access to financial assistance programs in the United States, however, often depends on an “emergency” classification [35,36]. This creates a barrier for chronic, vision-threatening conditions. The following case illustrates the consequences:

*The patient is a 72-year-old Nigerian man diagnosed with advanced open angle glaucoma and visually significant cataracts. He was seen during an EOW event at an FQHC and referred to the VI due to glaucoma. The ophthalmologist recommended glaucoma surgery within three months. His application for Medicaid was delayed. Hospital policy required confirmed insurance to proceed with surgery. The FQHC team at the host site initiated an Emergency Medical Assistance application. Failure to designate treatment as an emergency caused repeated denials of assistance. At one point, though financial assistance had been approved, the patient was not scheduled for surgery because financial assistance was not considered a type of insurance by the institution, and the condition was not deemed urgent. After several attempts at rescheduling over the course of two years, the patient had surgery. His vision was nevertheless irreversibly damaged due to the delay.*

This case demonstrates how rigid emergency-based classification systems can misrepresent the urgency of chronic, time-sensitive ophthalmic disease and contribute to preventable blindness.

A structured prioritization framework, such as a MeNTS-informed model, could better align scheduling with both clinical urgency and real-world patient vulnerability. Although existing versions do not incorporate SDOH, adding variables such as insurance instability, language barriers, limited transportation, and navigation challenges could improve equity and prevent harmful delays [26,27,28,29,30,31,32,33,34].

Glaucoma, diabetic retinopathy, neovascular AMD, and advanced cataract are all medically necessary, time-sensitive conditions. Prioritization tools adapted to reflect both clinical and social risk can help ensure that patients receive timely care and that avoidable vision loss does not occur simply because the system lacks an appropriate way to classify urgency.

*B*. ***Patient disengagement driven by cumulative structural and logistical barriers***

Terms such as “lost to follow-up,” “nonadherence,” and “noncompliance” are often used to describe patients who do not complete recommended evaluations or treatments. These labels obscure underlying causes and imply personal failure. Disengagement, by contrast, refers to a gradual loss of participation in care driven by structural, and relational, factors outside the patient’s control [14,15,16,17].

Multiple contributors to disengagement have been described in the literature, including transportation limitations, inflexible work schedules, unstable housing, inconsistent phone access, language discordance, and negative encounters with healthcare systems [37,38,39,40,41,42,43]. When the effort required to navigate care exceeds the perceived benefit, especially in diseases with slow or invisible progress, patients understandably begin to withdraw [14,15,16,17].

These pressures are intensified in underserved populations, where immediate needs such as housing, food, and employment take precedence over long-term health [2,3,4,5,26,27,28,29,30,31,32,33,34]. When healthcare systems appear rigid, inaccessible, or unresponsive, disengagement accelerates: calls go unanswered, appointments remain unscheduled, and applications for financial aid are abandoned. Systems may misinterpret this as “noncompliance,” reinforcing a cycle that is structurally driven rather than patient driven.

These patterns are particularly relevant to ophthalmology, where common chronic blinding conditions demand long-term engagement, but often offer no immediate symptom relief.

- Stable glaucoma generally requires 2 to 3 office visits each year and can require use of medication several times each day [8].
- Advanced diabetic retinopathy can require office visits every 2 to 3 months in addition to close attention to blood sugar control in the setting of visits to an internist, endocrinologist, podiatrist and nephrologist [10].
- The wet form of macular degeneration can require injections on a once/month basis at first and monitoring several times each year once stabilized [12].

For patients with poor vision, each appointment requires transportation coordination, financial sacrifice, and substantial time investment. When stability, meaning “nothing has changed”, is the expected outcome, it can be difficult to remain engaged. The following case illustrates how disengagement emerges not from lack of responsibility but from repeated encounters with barriers that make care unsustainable.

*A 75-year-old Hispanic woman was first seen at an EOW event in 2018 and referred for cataract surgery and glaucoma evaluation. Surgery was not scheduled due to funding limitations, and she pursued surgery abroad. She re-engaged in U.S. care in 2021 and underwent YAG capsulotomy, but persistent glaucoma suspicion required follow-up. A medically necessary blepharoplasty was recommended but classified as “non-urgent,” making it ineligible for insurance and institutional financial assistance. Despite eventually obtaining a Social Security number and financial aid approval, the procedure remained unscheduled. Repeated misunderstandings, communication challenges, and insurance-related obstacles ultimately led her to disengage from care entirely.*

Her experience demonstrates how multiple personal, financial, and logistical pressures accumulate until continued participation feels futile. While it is impossible to point to a single root cause for this patient’s disengagement, her experience demonstrates how barriers can accumulate until continued participation in care feels futile. Several strategies might be employed to avoid disengagement:

- **Ensure patient (and provider) comprehension**

Communication must go beyond basic translation. It is essential that the provider understands and addresses the patient’s concerns and that the patient understands the provider’s concerns. If professional interpreters are not available during first contact, arrangements need to be made for a subsequent facilitated conversation. Patients need to understand not just what they are being asked to do, but why it matters, particularly for asymptomatic conditions like glaucoma or diagnoses that require extended timelines to see improvement or stability, if they are to prioritize eye care in the face of more pressing and tangible health or personal concerns [4,5,44,45,46,47].

- **Assign a patient navigator to build continuity and trust**

A single, identifiable, accountable person who follows the patient is essential. Navigators should be able to schedule appointments, arrange transportation, assist with forms, facilitate contact with providers and agencies and help the patient interpret the next steps. More importantly, they build a sense of continuity and trust—a sense that someone knows them and will notice if they fall off track. This trusted relationship serves as the anchor that keeps a patient engaged in what would otherwise feel like an impersonal system [48,49,50,51,52].

- **Document and use the patient’s actual means of communication**

Many patients in vulnerable populations do not have reliable access to a personal cell phone. Contact information can change frequently. Systems should actively ask, document, and confirm the best method to reach the patient—whether that is a family member’s phone, a neighbor’s number, a preferred messaging app like WhatsApp, or contact via the patient’s primary care clinic [53,54]. Verifying contact information at every interaction rises to the importance of checking vital signs.

These proposals reflect an understanding that disengagement, regardless of cause, is a modifiable risk factor for poor outcomes.

*C*. ***Failures that arise when the healthcare system, functioning as a complex adaptive system (CAS), is unable to adapt to patients’ and systems’ changing circumstances.***

Health care delivery is inherently complex. It occurs within a network of interdependent agents including patients, providers, payers, and public and private systems. Each operates in its own environment and often lacks full awareness of what others are doing. Health systems researchers describe this as a Complex Adaptive System (CAS). In a CAS, the actions of one agent influence, and are influenced by, the actions of others. Failure rarely stems from a single event; rather, it results from cascading breakdowns in coordination, feedback, and adaptation [18,19,20,21].

CASs are common in nature and society. Examples include the immune system, termite colonies, the stock market, and airport terminals. In these systems, individual agents respond to local conditions without centralized control. Combined actions produce outcomes that are nonlinear, unpredictable, and emergent. An adaptive response in one part of the system may cause harm in another. For example, inflammation is protective at the local level but can damage healthy tissue when exaggerated. These dynamics illustrate how actions that make sense locally can have unintended negative consequences globally [55,56,57,58,59,60,61,62].

Health care functions in much the same way. It includes a constantly shifting set of relationships among patients, families, clinicians, hospitals, insurers, social services, policies, and technologies. These entities rarely coordinate in real time or under shared responsibilities. As a result, failure often comes not from a single mistake but from fragmentation, poor communication, and rigidity lacking adaptability [58,59,60,61,62]. Real-life circumstances change frequently, yet systems are unable to adapt. Individual agents are rarely equipped or permitted to adjust their decisions or workflows to meet those evolving needs.

This dynamic pose particular risks for vulnerable patients, as the rules governing charity programs, clinic scheduling, medical care, and immigration policy often shift in response to external factors such as funding and public policy [63,64]. When these rules are applied rigidly, the system loses its ability to adapt to individual circumstances, leaving many patients to fall through the cracks. This perspective is highly relevant to ophthalmology. Chronic conditions like diabetic retinopathy or glaucoma require long-term engagement with care. Even when the disease is stable, patients typically need several visits per year [8,10,12]. Each visit depends on aligning multiple elements including provider availability, access to diagnostic testing, translation services, transportation, and financial support. All of them must be in place for care to continue and for vision to be preserved.

The following case shows how a patient who started within a functioning care system ultimately experienced poor outcomes when that system failed to adapt.

*A 50-year-old uninsured Hispanic man was diagnosed in 2017 with end-stage post-traumatic chronic angle-closure glaucoma (CACG) in his left eye and primary angle-closure glaucoma (PACG) in his right eye following a traumatic eye injury in childhood. Initially, he received coordinated, high-quality care supported by Spanish interpreters, a donor-sponsored financial aid program, and scheduled follow-ups and procedures at the Vision Institute. However, when the donor program ended, he was left without financial coverage, which disrupted his care and follow-up. While he was ultimately able to apply for financial assistance with support from his FQHC, in the interim he was turned away from care twice due to lack of both insurance and an interpreter. He struggled to complete financial aid forms and missed appointments, leading to a loss of access to interpretation services and further disengagement from care. Multiple outreach attempts were unsuccessful. His citizenship status disqualified him from Medicare, and out-of-pocket costs were unaffordable. After two years of being lost to follow-up, he was finally approved for financial assistance and able to re-engage in care. By that time, his severe glaucoma had progressed, with vision worsening to 20/400, and he now requires additional surgical interventions that are still underway.*

This case exemplifies key features of CAS failure:

- **Lack of system awareness.** The expiration of the donor program was known, but no entity assumed responsibility for planning the transition.
- **Limited adaptability.** When circumstances changed, rigid protocols prevailed. Without insurance or funding, the patient became ineligible for continued care, regardless of clinical need.
- **Communication gaps**. Neither the clinical team nor the referring clinic knew the funding had ended. They were unaware of why the patient missed visits. Standard no-show procedures failed to account for language barriers or unstable contact information.
- **No redundancy or feedback loop**. Missed appointments were noted, but no alert or outreach was triggered. There was no mechanism to recognize that the system was beginning to fail.
- **Patient-level complexity ignored**. The patient’s immigration status, language needs, and limited health system literacy were no longer considered once structured support ended. The system defaulted to standardized expectations in a non-standard case.

No individual actor intended harm; rather, the structure itself lacked mechanisms to detect instability, absorb variation, and adapt to changing conditions.

As Leykum et al. have described, health systems must manage uncertainty across several dimensions: standardized versus customized care, interdependent workflows, and routine versus non-routine tasks [56]. In this case, the patient required customized care, yet rigid eligibility rules prevailed. Multiple organizations were involved, but none coordinated communication or follow-up. His needs including interpretation, navigation, and financial support met with inflexible processes.

Complexity science distinguishes between reducing uncertainty through standardization and absorbing it through contextual adaptation [62,65,66,67,68,69,70]. The patient required an adaptable system; instead, the system adhered to fixed rules. It failed to absorb variation, detect early signs of trouble, or maintain continuity once structured support disappeared.

WHO has emphasized the need for feedback loops and real-time responsiveness in complex systems [61]. Yet here, no feedback mechanism linked the patient’s disengagement to an adaptive response. The system could not see that it had failed.

Improving outcomes in complex systems requires more than stronger individual components; it requires better coordination, real-time awareness, and structural flexibility [58,59,60,61,62,65,66,67,68,69,70]. The following proposals focus on building adaptive capacity into care systems for vulnerable patients:

- **Anticipate instability**. Programs that rely on time-limited funding or external support should include an exit strategy and clear plans for transition. Patients who lose eligibility must be proactively identified and connected to alternative resources before a gap in care occurs [56,62,68]
- **Create feedback loops**. Missed appointments, changes in insurance status, or loss of communication should trigger alerts within the care system. These signals should be routed not just to schedulers but to those who can intervene including patient navigators and clinicians [53,71].
- **Embed navigation as infrastructure**. As noted above, patient navigators are essential. They are core members of the care team for high-risk patients. Navigators can track eligibility, communicate across silos, and assist with applications, transportation, and language access, especially when structured supports fall away [48,49,50,51,52,72].
- **Document and coordinate across systems.** A centralized record of the patient’s funding status, communication preferences, and navigation history can support continuity of care. This requires cooperation across referring clinics, specialty providers, and aid programs, and policies that support data sharing when appropriate.
- **Train for flexibility.** Staff at every level should be trained to recognize when a patient’s situation falls outside standard workflows, and be empowered to pause, escalate, or adjust when needed. Rigid adherence to rules must give way to problem-solving when lives or sight are at stake [65,66,67,68,69,70].

These proposals reflect a shift toward strengthening the relationships and responsiveness of the system. In a CAS, resilience depends not on perfect execution, but on the capacity to adapt when conditions change.

Table 2 presents the systemic and structural barriers identified in the thematic synthesis and organizes them according to the three analytic themes used in the manuscript.

## 4. Limitations

This narrative review has certain limitations inherent to its design. Because the goal was to integrate insights from the literature with real-world experience, the review does not aim to be comprehensive, and article selection emphasized relevance to the thematic framework rather than exhaustive coverage. The illustrative cases were purposively selected from a single mobile eye care program to highlight distinct systemic patterns. While these cases reflect barriers commonly encountered across underserved populations, they do not represent the full range of patient experiences in all settings. Finally, case narratives were reconstructed from de-identified program documentation and may not capture every contextual detail. Despite these limitations, the consistency between published evidence and the patterns observed in programmatic experience supports the robustness of the themes identified.

## 5. Conclusions

Underserved patients experience many obstacles when trying to access eye care, but our cases show that preventable vision loss often results from how the healthcare system fails to adapt to their needs rather than from patient behavior alone. We identified three interconnected contributors: serious eye diseases being treated as “non-urgent,” cumulative barriers that lead patients to disengage, and breakdowns in coordination across the care system. These problems reflect a maladaptive complex adaptive system, one in which processes are fragmented, communication is limited, and rigid rules prevent the system from adjusting when patient circumstances change. These patterns were most clearly observed in the patient population represented in our cases and may manifest differently in other demographic or geographic groups. Improving equity in eye care will require better coordination, stronger patient navigation, and prioritization frameworks that account for both clinical urgency and social context. Building a more flexible and responsive system is essential to preventing avoidable vision loss.

## Figures and Tables

**Table 1:** Differential Impact of systemic Barriers on Major Ophthalmic Diseases.

Disease	Why Time Matters	Required Timing of Care	Most Relevant Barriers	Impact of Delayed Care	Illustrative Connections to Cases	Supporting Literature
Glaucoma	Often asymptomatic until advanced; progressive optic nerve damage; irreversible visual field loss.	Months (progressive if chronic, acute hours)	Misclassification as “non-urgent”; delays in surgery authorization; missed visits; lack of insurance coverage	Permanent loss of vision; worsening cupping; reduced functional independence	Case A: 2-year delay in glaucoma surgery due to incorrect classification and insurance obstacles led to irreversible vision loss.	[6,7,8]
Diabetic Retinopathy (DR)	Rapid progression in advanced stages; high benefit from timely anti-VEGF, laser, and metabolic control	Weeks-months (depends on severity)	Insurance delays for treatment; administrative complexity; transportation instability; fragmented care between specialist (PCP-Ophthalmology-Endocrinology); patient education.	Worsening edema; hemorrhage; tractional detachment; irreversible vision loss	Relevance highlighted in Results to show time-sensitive harm when follow-up lases occur.	[9,10]
Age-Related Macular Degeneration (AMD)-Advanced stages	Requires strict injection intervals to preserve vision; rapid decline with even short delays	Weeks (if advanced), Months-years if early stages	Financial hardship, transportation issues to repeat injections, insurance lapses, scheduling bottlenecks; patient education.	Permanent central vision loss from delayed injections; worse functioning outcomes; reduced independence.	Addressed conceptually where misclassification disproportionately harms time-sensitive but non-emergent conditions.	[11,12]
Cataract	Not emergent but crucial for function, driving, working and independence; delays affect quality of life and may cause falls, accidents, and affect functionality or cause other eye diseases such as lens-induced glaucoma (phacomorphic glaucoma)	Variable (but functionally disabling)	Insurance classification as “elective”; long wait times to get financial support; immigration/insurance support	Prolonged functional impairment; risk of falls; loss of independence; difficulty meeting other medical needs; risk of other eye diseases.	Case B: cataract-related disability compounded by deferred surgery due to financial/insurance classification barriers.	[13]

This table summarizes how major ophthalmic diseases differ in their time sensitivity, vulnerability to systemic barriers, and potential consequences of delayed care. The comparisons are based on established patterns reported in the literature and are intended to illustrate relative differences rather than define strict prognostic timelines.

**Table 2:** Summary of Structure/Systemic Barriers identified across themes.

Analytic Theme	Description	Type of Barrier	System-Level Mechanisms	Examples of System Dysfunction	Clinical Implications	Illustrative Cases
A. Misclassification of Medically Necessary, Time-Sensitive Care	Vision-threatening chronic diseases are often labeled “non-urgent,” preventing timely intervention.	Administrative classification; insurance eligibility; financial aid policies	Binary “emergency vs. elective” decisions fail to capture chronic vision threatening diseases requiring interventions	Medicaid delays; denied scheduling of procedures due to lack of “urgent” designation; postponement of medically necessary surgery	Progression of disease while waiting for approval; irreversible vision loss due to delayed intervention	Case A: Advanced glaucoma patient whose surgery was delayed 2 years due to incorrect urgency classification and insurance barriers
B. Patient disengagement Driven by Structural and Logistic Barriers	Patients appear “noncompliant”, but disengagement reflects cumulative burden.	Language access; documentation demands; multi-step navigation processes for insurance, diagnosis, treatment and surgery scheduling; loss of communication	Complex language in processes related to insurance application and scheduling; Care becomes too difficult to complete; overwhelming administrative load; lack of clear communication	Missed appointments due to misunderstanding; inability to complete financial assistance applications; misinterpretation of provider recommendations	Missed visits, gaps in follow-up, worsening diseases attributed incorrectly to patient behavior	Case B: patient who travelled abroad for cataract surgery, later disengaged when surgery for dermatochalasis was label as not-urgent and repeatedly postpone
C. Complexity and Cascading Failures in a Complex Adaptative System (CAS)	Healthcare behaves as a interconnected system where small failures can amplify across actors	Fragmentation across clinics, insurers, aid programs, other providers; loss of coordination when changes happen	System cannot adapt to changes (insurance shifts, funding expiration, contact instability)	Patient dropped from care after donor program ends; no alerts when contact number fails; no system-level follow-up o no-shows	Minor administrative changes trigger major disruptions in care pathway; avoidable deterioration in vision	Case C: Patient with trauma-related glaucoma who lost access after donor funding ended; missed appointments triggered no intervention putting him at risk for disease progression.

This table presents the systemic and structural barriers identified in the thematic synthesis and organizes them according to the three analytic themes used in the manuscript. The table focuses on system-level mechanisms and pathways of failure and is not intended to describe disease-specific outcomes.

## Data Availability

No new data were created or analyzed in this study. Data sharing is not applicable to this article.

## Abbreviations

The following abbreviations are used in this manuscript:

EOW	Eyes on Wheels
FQHC	Federally Qualified Health Centers
CAS	Complex Adaptive System (CAS)
VI	Vision Institute
MeNTS	Medically Necessary, Time-Sensitive Score
SDOH	Social Determinants of Health
PCO	Posterior Capsular Opacity
OD	Oculus Dexter (right eye)
OS	Oculus Sinister (left eye)
IOP	Intraocular pressure
OCT	Optical Coherence Tomography
RNFL	Retinal Nerve Fiber Layer
CACG	Chronic Angle Closure Glaucoma
PACG	Primary Angle Closure Glaucoma

## Appendix A

**Table A1:** Search strategy for: Through Lens of complexity: A Narrative Review of Systemic Barriers to Eye Care.

Search Theme	Database Used	Search Terms	Number of Articles Screened (Pubmed/Web of Science/Google Scholar)
Healthcare Disparities & Insurance Navigation	PubMed, Web of Science	(“Healthcare Disparities”[Mesh] OR “healthcare disparit*”[Title/Abstract] OR “health care disparit*”[Title/Abstract] OR “healthcare inequit*”[Title/Abstract]) AND (“Ophthalmology”[Mesh] OR ophthalmology[Title/Abstract] OR ophthalmic[Title/Abstract]) AND (“Patient Navigation”[Mesh] OR “Insurance, Health”[Mesh] OR “Patient navigat*”[Title/Abstract] OR “Federally Qualified Health Center*”[Title/Abstract] OR Insurance[Title/Abstract])/((ALL=((healthcare disparit* OR health care disparit* OR healthcare inequit*)AND ((ALL=((ophthalmology OR ophthalmolog* OR ophthalmic*))AND ((ALL=((patient navigat* OR federally qualified health center* OR insurance))	36/162
General Vision & Insurance Coverage (U.S.)	PubMed, Web of Science, Google Scholar	(“Ophthalmology”[Mesh] OR “Eye Diseases”[Mesh] OR “Vision Disorders”[Mesh] OR “eye disease”[tiab] OR “eye diseases”[tiab] OR “eye disorder”[tiab] OR “eye disorders”[tiab] OR “vision care”[tiab] OR “eye care”[tiab]) AND (“Insurance, Vision”[Mesh] OR “insurance coverage”) AND (“United States”[Mesh])/((ALL=((“ophthalmology” OR “eye disease” OR “eye diseases” OR “eye disorder” OR “eye disorders” OR “vision care” OR “eye care” OR “vision disorder” OR “vision disorders”))) AND ALL=((“vision insurance” OR “insurance coverage”))) AND ALL=((“United States” OR USA OR “U.S.”))/(“ophthalmology” OR “eye disease” OR “eye diseases” OR “eye disorder” OR “eye disorders” OR “vision care” OR “eye care”) AND (“vision insurance” OR “insurance coverage”) AND (“United States” OR USA OR “U.S.”)	115/103/16,800
Complexity in Healthcare Navigation	PubMed	((Complexity) AND (Healthcare)) AND ((“Ophthalmology”[Mesh] OR “Eye Diseases”[Mesh] OR “Vision Disorders”[Mesh] OR “eye disease”[tiab] OR “eye diseases”[tiab] OR “eye disorder”[tiab] OR “eye disorders”[tiab] OR “vision care”[tiab] OR “eye care”[tiab]))	733
Social Determinants of Health (SDOH)	PubMed, Web of Science	(“Ophthalmology”[Mesh] OR “Eye Diseases”[Mesh] OR ophthalmology[tiab] OR ophthalmic[tiab] OR “eye care”[tiab] OR “eye condition”[tiab] OR “eye conditions”[tiab] OR “eye disease”[tiab] OR “eye diseases”[tiab] OR “eye health”[tiab] OR “vision care”[tiab] OR “vision health”[tiab]) AND (“Social Determinants of Health”[Mesh] OR “social determinant”[tiab] OR “social determinants”[tiab] OR SDOH[tiab] OR “social factor”[tiab] OR “social factors”[tiab] OR “social need”[tiab] OR “social needs”[tiab] OR “social stress”[tiab] OR “social stressor”[tiab] OR “social stressors”[tiab])/(ALL=((“ophthalmology” OR ophthalmic OR “eye care” OR “eye condition” OR “eye conditions” OR “eye disease” OR “eye diseases” OR “eye health” OR “vision care” OR “vision health”))) AND ALL=((“social determinants of health” OR “social determinant” OR “social determinants” OR SDOH OR “social factor” OR “social factors” OR “social need” OR “social needs” OR “social stress” OR “social stressor” OR “social stressors”))	72/305
MeNTS Score in Ophthalmology	PubMed, Web of Science	(“Ophthalmology”[Mesh] OR “Eye Diseases”[Mesh] OR “Vision Disorders”[Mesh] OR “eye disease”[tiab] OR “eye diseases”[tiab] OR “eye disorder”[tiab] OR “eye disorders”[tiab] OR “vision care”[tiab] OR “eye care”[tiab]) AND (“Time”[Mesh] OR “Time-to-Treatment”[Mesh] OR “Operative Time”[Mesh] OR “Time Factors”[Mesh] OR “Emergencies”[Mesh] OR “Emergency Treatment”[Mesh] OR “Emergency Service, Hospital”[Mesh] OR “Emergency Use Authorization”[Mesh])/(ALL=((“ophthalmology” OR “eye disease” OR “eye diseases” OR “eye disorder” OR “eye disorders” OR “vision care” OR “eye care”))) AND ALL=((time OR “time to treatment” OR “operative time” OR “time factors” OR emergencies OR “emergency treatment” OR “emergency service, hospital” OR “emergency use authorization”))	307/651
Definitions of Emergency & Urgency in Ophthalmology	PubMed, Web of Science	(“Ophthalmology”[Mesh] OR “Eye Diseases”[Mesh] OR “Vision Disorders”[Mesh] OR “eye disease”[tiab] OR “eye diseases”[tiab] OR “eye disorder”[tiab] OR “eye disorders”[tiab] OR “vision care”[tiab] OR “eye care”[tiab]) AND (“Time”[Mesh] OR “Time-to-Treatment”[Mesh] OR “Operative Time”[Mesh] OR “Time Factors”[Mesh] OR “Emergencies”[Mesh] OR “Emergency Treatment”[Mesh] OR “Emergency Service, Hospital”[Mesh] OR “Emergency Use Authorization”[Mesh])/(ALL=((“ophthalmology” OR “eye disease” OR “eye diseases” OR “eye disorder” OR “eye disorders” OR “vision care” OR “eye care”))) AND ALL=((time OR “time to treatment” OR “operative time” OR “time factors” OR emergencies OR “emergency treatment” OR “emergency service, hospital” OR “emergency use authorization”))	26,873/651
